# 

**Published:** 1966-01

**Authors:** 


### BRISTOL MEDICO-CHRURGICAL JOURNAL

Incorporating
The Medical Journal of the South West

Editors :
N. J. BROWN, M.B., M.R.C.P.
A. B. RAPER, M.D., F.R.C.P.

1966 *VOL.* 81

Published quarterly under the auspices of the
Bristol Medico-Chirurgical Society.

Printed by
F. Bailey & Son Ltd., Dursley, Glos.

THE

### BRISTOL MEDICO-CHIRURGICAL JOURNAL

### 1966 CONTENTS OF VOL. 81, Nos. 299 - 302

| | | Page |
|---|---|---|
| Society, Artists and the Plague | R. E. Hemphill | 1 |
| Milk and Tuberculosis | A. D. Osborne and A. V. Neale | 12 |
| Unilateral Renal Arterial Obstruction | Clinico-Pathological Conference | 15 |
| Wellcome Trust Grant to the Department of Biochemistry, University of Bristol | | 21 |
| Tumours of the Skin | D. C. Bodenham | 23 |
| Serological Tests for Syphilis | H. R. Cayton | 32 |
| The Biochemical Detection of Inherited Diseases | J. B. Holton | 35 |
| The Evolution of Liver Surgery | A. G. Riddell | 39 |
| Advances in the Management of Rh Hæmolytic Disease | Geoffrey H. Tovey | 46 |
| Viruses and Cancer | Edward S. Meek | 52 |
| Brook's Family Herbal | A. P. Radford | 55 |
| The Advance of Gastroenterology | J. M. Naish | 59 |
| Intimations of Senility | T. W. Lloyd | 66 |
| Experiences in Control of Anti-coagulant Therapy | A. B. Raper | 69 |
| The History of Jaundice in Bristol | Bimla Arora | 74 |

THE

### BRISTOL MEDICO-CHIRURGICAL JOURNAL

*(Incorporating the Medical Journal of the South-West)*

Established 1883

VOL. 81 (i) | January 1966 | NO. 299

### *Contents*

SOCIETY, ARTISTS, AND THE PLAGUE *R. E. Hemphill* 1

MILK AND TUBERCULOSIS *A. D. Osborne and A. V. Neale* 12

UNILATERAL RENAL ARTERIAL OBSTRUCTION
*Clinico-Pathological Conference* 15

NOTES AND NEWS . . . . . . . . . . 21

*PUBLISHED QUARTERLY*

*ANNUAL SUBSCRIPTION* 16/- *POST FREE*

BRISTOL MEDICO-CHIRURGICAL JOURNAL

PUBLISHED UNDER THE AUSPICES OF THE

### *Bristol Medico-Chirurgical Society*

The Society was founded in 1874. In 1883 publication of this Journal began, a[illegible] has continued quarterly since then. During the years 1949 to 1962 it appeared und[illegible] the title of "The Medical Journal of the South West".

The Society founded a medical library in 1890, which in 1892 was combined wi[illegible] that of the Bristol Medical School. This became the University of Bristol Medi[illegible] Library in 1925, and an agreement in that year confirmed the privilege of Members [illegible] use the University Medical Library.

### *Editorial Committee*

Dr. N. J. Brown, Southmead Hospital, Bristol. *Joint Editor.*

Dr. A. B. Raper, Department of Pathology, Bristol Royal Infirmary. *Joint Editor.*

Dr. J. Macrae, Ham Green Hospital.

Dr. R. C. Wofinden, Central Clinic, Bristol.

Mr. A. E. S. Roberts, Medical Library, University of Bristol. *Secretary.*

*Local Correspondents*

*Bath* . . Mr. A. D. Bateman, 3 The Circus, Bath.

*Exeter* . . Dr. L. N. Jackson, Mount Jocelyn, Crediton, Devon.

*Gloucester* . Dr. R. F. Jarrett, 9 College Green, Gloucester.

*Plymouth* . Dr. C. R. Croft, Lexden, Hartley Av., Plymouth.

*Taunton* . Dr. M. McAlley, 1 The Crescent, Taunton.

*Torquay* . Dr. A. Robinson Thomas, 20 Devon Square, Newton Abbot, Devon.

*Truro* . . Dr. F. D. M. Hocking, St. Andrews, Perranporth, Cornwall.

*Yeovil* . . Mr. J. A. Vere Nicoll, Knapp House, Yeovil, Somerset.

### NOTICE TO CONTRIBUTORS

Original articles are invited, provided they have not been published elsewhere. Notes of forthcoming events, meetings held, degrees conferred, staff appointments, and other local news are welcomed. The editorial committee reserves the right to make literary corrections.

Illustrations should always be supplied ready for reproduction. All re-touchings or other operations required will be charged to the author. Line drawings should be drawn in Indian ink. We regret that we must ask authors to pay for making blocks, if this is necessary.

J. W. ARROWSMITH LTD., WINTERSTOKE ROAD

BRISTOL.

### Notes and News

### £12,000 GRANT TO BRISTOL UNIVERSITY FITS OUT LABS FOR BIOCHEMICAL STUDY

A grant of £12,000 from the Wellcome Trust will be used to adapt existing research accommodation to provide an extension for the University of Bristol's Department of Biochemistry. It is hoped that alterations will begin in the autumn.

Professor P. J. Randle, Head of the Department of Biochemistry, is to use the additional 12,000 square feet of laboratory space for the development of research in molecular biology and physical biochemistry. It is envisaged that the unit will ultimately provide accommodation for some 20 to 24 additional research workers.

The Wellcome Trust was created by the will of the late Sir Henry S. Wellcome, who died in 1936. It is currently making grants at the rate of £1 million a year for medical and allied research.

### UNIVERSITY OF EDINBURGH MEDICAL SCHOOL CREATES THREE TROPICAL MEDICAL LECTURESHIPS

Using funds offered by the Ministry of Overseas Development, the University of Edinburgh is to create at its Medical School two senior and one junior lectureships in the field of tropical medicine.

The senior lectureships will enable selected doctors and research workers with special experience of preventive and curative medicine in the tropics to continue to work in this field. Lecturers appointed under this scheme will form a pool of talent which can be utilized for teaching and research work at home and overseas, either in association with the technical assistance programmes through which the Ministry of Overseas Development gives aid to developing countries, or by secondment to overseas universities, hospitals and medical research institutions. They will also help to train in their special subjects younger men and women wishing to specialize in some branch of tropical medicine, who may be appointed to a junior lectureship.

The lectureships will be open to British subjects of the United Kingdom with the necessary qualifications, and who are willing to serve overseas on secondment. Eighteen similar lectureships at the London and Liverpool Schools of Tropical Medicine have been financed by the Ministry of Overseas Development.

Progress in the prevention and cure of disease in tropical countries must be based on experience and research in such specialized subjects as parasitology, entomology, microbiology and tropical hygiene, and in the treatment of conditions peculiar to the tropics. In the knowledge and development of these subjects Britain has in the past taken a leading place which can be maintained only by those who have worked for long periods in tropical areas. Many of those so qualified who can supply the requisite medical and research experience are now returning to this country, however, and if they now turn to other branches of medicine, their special skills will be lost to their subjects.

### SPECIAL COMMONWEALTH AWARD FOR EDINBURGH PHYSICIAN

Mrs. Barbara Castle, the Minister of Overseas Development, has made a Special Commonwealth Award of £1,500 a year to Sir James Cameron who is to be Professor of Medicine at Dacca Medical College in Pakistan. Sir James, who received a knigh[illegible] hood in the recent Birthday Honours, is Senior Consultant Physician to the Ro[illegible] Infirmary, Edinburgh.

Special Commonwealth Awards, which are additional to the normal salary a[illegible] allowances of the post, are available on the advice of the Committee for Univers[illegible] Secondment, to eminent British academics going, for at least two years, to take [illegible] important posts in universities in developing Commonwealth countries.

Sir James is Senior Consultant Physician to the Royal Infirmary, Edinburgh, a[illegible] a member of the clinical teaching staff of the University of Edinburgh. From 1926–[illegible] he was a lecturer in Physiology at the University of Edinburgh and from 1927–38 [illegible] was consultant at the Royal Public Dispensary. From 1941–44 he was consult[illegible] physician to Southern Command, India, and from 1944–45 to the General Hea[illegible] quarters, India and Burma. On leaving the army Sir James was appointed Honora[illegible] Consultant to the India and Burma Offices.

Sir James is a member of the Edinburgh Post-graduate Board for Medicine, and [illegible] the Scottish Post-graduate Association. From 1960–63 he was President of the Ro[illegible] College of Physicians, Edinburgh. In 1963–64 he made an extensive study visit [illegible] Pakistan, India, Burma and Ceylon. His special interest is in abdominal medicine [illegible] gastro-enterology, and hepatic and renal disease.

*Printed by*

J. W. Arrowsmith Ltd., Bristol

